# Recruitment rates and reasons for community physicians' non-participation in an interdisciplinary intervention study on leg ulceration

**DOI:** 10.1186/1471-2288-9-61

**Published:** 2009-08-14

**Authors:** Oliver R Herber, Wilfried Schnepp, Monika A Rieger

**Affiliations:** 1 Institute of General Practice and Family Medicine, Faculty of Medicine, University of Witten/Herdecke, Alfred-Herrhausen-Str. 50, 58448 Witten, Germany; 2 Department for Family-oriented and Community-based Nursing, Institute of Nursing Science, University of Witten/Herdecke, Stockumer Str. 12, 58453 Witten, Germany; 3 Institute of Occupational and Social Medicine, University Hospital Tübingen, Wilhelmstr. 27, 72074 Tübingen, Germany

## Abstract

**Background:**

This article describes the challenges a research team experienced recruiting physicians within a randomised controlled trial about leg ulcer care that seeks to foster the cooperation between the medical and nursing professions. Community-based physicians in North Rhine-Westphalia, Germany, were recruited for an interdisciplinary intervention designed to enhance leg ulcer patients' self-care agency. The aim of this article is to investigate the success of different recruitment strategies employed and reasons for physicians' non-participation.

**Methods:**

The first recruitment phase stressed the recruitment of GPs, the second the recruitment of specialists. Throughout the recruitment process data were collected through phone conversations with GP practices who indicated reasons for non-participation.

**Results:**

Despite great efforts to recruit physicians, the recruitment rate reached only 26 out of 1549 contacted practices (1.7%) and 12 out of 273 (4.4%) practices during the first and second recruitment phase respectively. The overall recruitment rate over the 16-month recruitment period was 2%. With a target recruitment rate of n = 300, only 45 patients were enrolled in the study, not meeting study projections. Various reasons for community physicians' non-participation are presented as stated spontaneously during phone conversations that might explain low recruitment rates. The recruitment strategy utilised is discussed against the background of factors associated with high participation rates from the international literature.

**Conclusion:**

Time, money, and effort needed during the planning and recruitment phase of a study must not be underestimated to avoid higher than usual rates of refusal and lack of initial contact. Pilot studies prior to a study start-up may provide some evidence on whether the target recruitment rate is feasible.

**Trial registration:**

Current Controlled Trials ISRCTN42122226.

## Background

### Treatment of Leg Ulcers in Germany

Unlike the UK, for instance, where common leg ulcers are diagnosed, treated, and followed up in nurse-run leg ulcer clinics, in Germany these patients are solely in the hands of physicians. Traditionally, the German health care system has no gatekeeping functions; instead patients are free to select a doctor of their choice (GP or specialist) who is located in the community. Since there is no mechanism to control this "self-selected" gatekeeping, patients frequently choose specialists directly. There are only a few incentives for patients to first contact a GP in Germany; none of them apply to leg ulcer patients. For German GPs it is financially rather unattractive to treat leg ulcer patients as the entire treatment has to be financed from the physicians overall budget, which ranges between 32 and 45 Euro per quarter and patient, depending on the federal state. Thus, the more often leg ulcer patients' visit their GP and the more material (e.g. wound dressing) they need, the greater the risk of budgetary overspending.

### Design of the Parent Study

Due to the chronic nature of venous leg ulceration, patients require coordinated, consistent, and collaborative multidisciplinary care to meet their specific daily needs associated with the disease which cannot be delivered through occasional visits to a physician's practice. Therefore an evidence-based, nurse-led education programme on leg ulceration that aims to enhance patients' self-care agency was developed. The educational programme was embedded in an open, multi-site clinical trial comparing healing rates, wound size, and health-related quality of life (HRQoL) indicators (ISRCTN42122226). The study was conducted in the federal state of North Rhine-Westphalia, Germany. The specific objective of the parent study was to compare healing rates, wound size and HRQoL in patients with venous leg ulcers whose care was (1) provided by a physician and a liaison nurse specialist (= tandem practice) with those whose care was (2) provided by a physician alone in order to assess the value of a nurse-led education programme. The approach of this programme was designed to actively involve patients and their dependent care agents in leg ulcer treatment through regular educational visits by nurses in order to increase the patients' understanding of their condition, care and contribution towards the success of the treatment. The intervention encompassed the presence of a nurse specialist educating patients and their relatives in participating physicians' practices or at patients' homes about leg ulcer related self-care activities for a maximum of 1 year or earlier until the complete healing of the wound occurred. An evidence-based self-care activity catalogue containing leg ulcer-related self-care actions for patients with venous leg ulcers was used as a reference list for nurse specialists to guarantee the conveyance of the same information to all patients. Scheduled appointments took place every fortnight within the first 2 months after the patient's enrolment in the study and thereafter once a month. During each 45-minute visit nurse specialists carefully selected one or more appropriate items from the catalogue depending on the patient's or carer's level of comprehension. Continued home visits were made by the same nurse to establish a nurse-patient relationship of mutual trust that is vital for patients with a chronic disease [1].

Physicians, who did not receive any monetary incentive for study participation, were asked to participate in the trial and to recruit leg ulcer patients from their practices. A number of steps were taken prior to the study start-up (modelling phase) in order to increase the willingness of physicians to participate. The study protocol was (1) reviewed with a practising GP and (2) discussed intensively in the quality circle of GPs cooperating with our Institute of General Practice and Family Medicine. To underline the interdisciplinary approach, the study was equally conducted by members of staff from general medicine and nursing. Finally, the principal investigator of the study was involved in a task force of a GPs-network planning to establish an integrated care system on the subject of leg ulceration. All requests for change were introduced into the study proposal. Ethical approval was obtained from the University of Witten/Herdecke Research Ethics Committee. All GPs' and - after amendments to the study protocol - dermatologists' and phlebologists' practices within the study area were eligible to participate in the study if they (1) were to be operational for the whole study period and (2) had at least one leg-ulcerated patient. Within the study area roughly 30 000 practices were potentially eligible for recruitment. Therefore, a pragmatic and geographic convenience sample of practices was drawn preferring practices in regions where contacts to nurse specialists had already been established by the researcher over the phone. After obtaining informed consent from the participating patients through a trained research assistant they were either linked up with a nurse specialist (intervention practice) or an independent assessor (control practice). Practice participation requirements were limited to affixing a patient identification badge (containing name, date of birth, postal address and phone number) on a pre-prepared fax form for referring patients. Interference with physicians' day-to-day clinical work was kept to a minimum. The physician-researcher relationship was maintained through ongoing personal contacts with the practices and recognition of the value of the physician's time. Periodic mailings were sent to participating practices as a reminder to continue enrolling patients and to provide information on the study's progress. We also established rapports with physician assistants in order to maximise participation and ease of contact with the physicians. Participating practices received chocolate bars, a voucher for a bouquet of flowers and a Christmas card for encouragement and thanks. At the end of the study all physician assistances' were offered a catch-up seminar in leg ulcer treatment as a non-monetary incentive for the practices. The efforts to maintain the physician-researcher relationship were of paramount importance as Germany still has little research tradition in general practice and GPs are not used to being participants in research [2].

### Aim of this paper

The physician recruitment was characterised by low participation rates. Obtaining voluntary participation of physicians is a widespread problem in health services research as international literature demonstrates [3-5]. Low participation rates in research projects are often a barrier to the completion of an otherwise well-designed project [6]. The aim of this paper is to report on (1) the recruitment rate in relation to the recruitment strategy employed and (2) to identify reasons for community physicians' non-participation in the clinical trial.

## Methods

### Recruitment process

Recruiting community-based practices (group or single) involved the identification of eligible practices and motivating them to participate. Due to the large amount of potentially eligible practices, the recruitment took place by phone. The first step involved the contacting of the practices and requesting involvement in the study. The recruitment coordinator (OH), a nursing science researcher, solicited participation from physicians by phone ("cold call"). A script was used to explain the study in a standardised way. Although the recruiter sought contact with leading physicians the calls were taken by physician assistants (administrative staff) in almost all cases. In such cases the strategy was to gain permission for the sending of an informational fax to be passed on to the physician. The fax consisted of a study outline and a five-item practice profile questionnaire for screening practice eligibility. All practices were asked to complete the questionnaire irrespective of participation in order to obtain information relevant to documenting a possible non-response bias. Usually a new arrangement for another phone appointment was made, yet often numerous calls were necessary before a contact with the physician could be established. If interest was signalled, physicians were asked to sign a participation agreement. Often physicians did not return this agreement so that close follow-up contacts were necessary. Finally a clinical group meeting was held for interested physicians at the university on a date accommodating their time schedules. The purpose of the meeting was to describe the study details, to answer questions, and to take advice from participating staff. Continuing medical education (CME) credits were provided to encourage physician attendance.

Alternatively an information package on the study procedure was sent to the physicians not attending the meeting.

#### First recruitment phase

The first recruitment phase targeting GPs' and dermatologists' practices took place between June and December 2005. Several recruitment strategies were employed using separate contact lists in order to document the recruitment process. The simplest strategy was to establish contacts with practices already cooperating with the University of Witten/Herdecke. Further potential practices were identified through the web pages of the Association of Compulsory Health Insurance (CHI) Physicians, Westphalia-Lippe and North Rhine. Additional GPs and dermatologists were retrieved through searches browsing Yellow Pages^®^. We used the topic search term 'GP' and 'dermatologist' and selected a radius of 50 kilometres around the locations of the nurse specialists. The latter also provided a list of physicians in their neighbourhood which they thought would be interested in participating. Finally, endorsement was sought from various professional societies such as the Association of Dermatologists, the Association of GPs Westphalia-Lippe and North Rhine and the German Society of Wound Healing and Wound Management (DGfW). In all cases we contacted the chairpersons asking for support getting physicians interested to participate or to advertise the study on their internal mail-server.

#### Second recruitment phase

The low recruitment rates of GPs and dermatologists' practices during the first recruitment phase made the development of a contingency plan necessary. It involved the launching of a second recruitment phase which took place from March until September 2006. The recruitment process was similar to the one mentioned above, but with two modifications. Firstly, we decided to include phlebologists since a large number of leg-ulcer patients in Germany are cared for by these specialists. Secondly, we refrained from obtaining written practice characteristics as the rate of return was very low. Instead practice eligibility was assessed over the phone. For the identification of phlebologists the Yellow Pages^® ^were searched and practices were selected within a radius of 50 kilometres around the nurse specialists' places of residence. In addition, the directory of the professional society of phlebologists was used.

### Analysis of recruitment process

**Figure 1:**
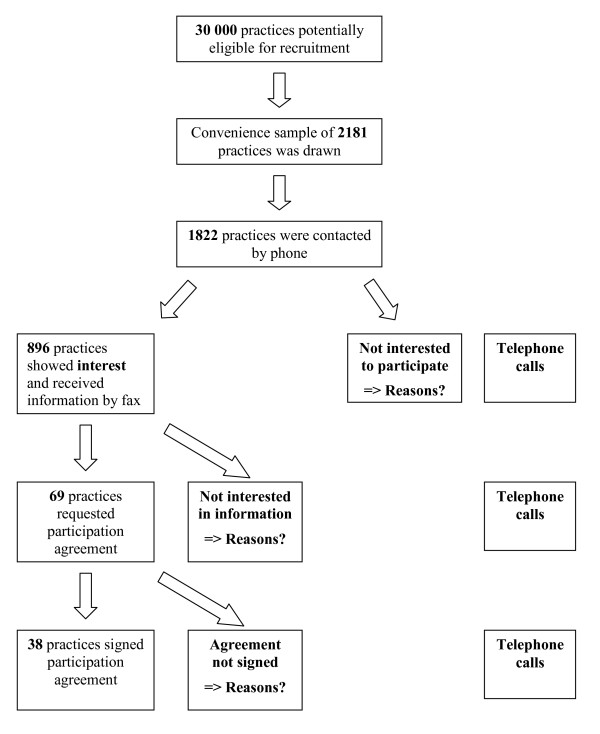
**Flowchart of recruitment process and assessment of reasons for non-participation**. The flowchart provides an overview of the recruitment process and an assessment of reasons for community physicians' non-participation.

Throughout the entire recruitment process over the phone data were collected about the recruitment encounters. Contact lists were fed into a computer system including the annotations made by the recruiter. The data were processed using MSExcel spreadsheet. The file contained the following items: (1) name of physician, (2) medical speciality, (3) number of phone calls made, (4) permission for faxing written information obtained (y/n), (5) participation agreement sent, and (6) participation agreement signed. Additionally reasons for community physicians' non-participation were recorded as stated by the practices throughout the entire recruitment process (Figure 1). To gain a deeper understanding of the reasons for non-participation we additionally contacted a manageable number of nine physicians by phone upon completion of the recruitment. Non-participating practices were randomly chosen from the MSExcel spreadsheet and contacted again. After several attempts all nine contacted physicians were available for a short phone conversation (Figure 1). Physicians - not physician assistants - were read out the same study outline as previously and were specifically asked to mention reasons crucial for their non-participation in the first place. As a prompt the interviewer mentioned that the research team was seeking to identify reasons for community physicians' non-participation in the study as groundwork for further research endeavours. The recruiter took detailed notes of the conversation after each encounter and wrote down reasons for refusal as stated spontaneously by the physician during the phone conversation. Originally, additional phone conversation data were sought to be analysed using content analysis. The conversations with the GPs turned out to be very short in length generally, making it impossible to analyse them by the use of content analysis [7]. The basic principles of Mayring's content analysis - just as any other form of content analysis - encompasses techniques such as units of analysis, step models and working with categories which cannot be implemented if interview data are of such short supply.

## Results

### Recruitment rates

During the first recruitment phase we retrieved 1875 listings of which 1549 were phoned. In 741 out of these 1549 cases (47.8%) the practice gave permission for sending a fax. Out of those, 105 practices completed - all or partly - the practice characteristics questionnaire. Merely 47 practices showed interest in participation. In the end 26 practices participated of which 19 were GPs and 7 dermatologists. This equals a recruitment rate of 1.7%. During the second recruitment phase 273 out of 306 practices were contacted of which 155 (56.7%) gave permission for sending a fax. Out of these 155 practices 24 requested a participation agreement. Finally, 12 practices participated, resulting in a recruitment rate of 5.1% for the second recruitment phase. Combining the results of the two recruitment phases, the response to the 1822 phone calls yielded 38 participating practices. Thus, the combined recruitment rate of the study was 2.0%.

Of all practices contacted 1412 (77.5%) were GPs, 371 (20.4%) were dermatologists and 39 (2.1%) were phlebologists. The results of the two recruitment phases are presented in the Additional file 1.

Physician assistants were answering almost all phone calls. Regarding the contact frequency, 75.9% of practices (1383) were contacted once, 17.1% (312) were contacted twice, and the remaining 7.0% (127) were contacted three times or more. The participation rate was 1.6% (23/1412) for GPs, 2.4% (9/371) for dermatologists, and 15.4% (6/39) for phlebologists. Thus, the recruitment rate was 1.6% for GPs and 3.6% for specialists (dermatologists, phlebologists). Physician participation at the clinical group meeting was low. Most of the interested physicians preferred an information package to physical attendance. Only five physicians attended the meeting of which only two finally participated in the study. Among the different strategies employed for recruiting GPs the contacting of the university's cooperation practices was the most successful strategy with a recruitment rate of 9.1%. The professional association endorsement strategy did not yield any practice participation. While both Associations of CHI Physicians could not offer any support, the DGfW agreed to help by promoting the study amongst their members, yet without yielding any physician participation.

### Reasons for Non-Participation

Reasons for non-participation were recorded throughout the entire recruitment process over the phone. In addition 9 physicians who did not participate in the study were called later. These additional phone conversations with GPs were rather limited with regard to data collection not yielding other reasons for non-participation apart from what was already known through the first recruitment encounter. Generally, physicians did not get involved in comprehensive interviews, phone calls lasted no longer than a minute with physicians stating spontaneously reasons for not participating in research. Since the information given was so limited, only data analysis using qualitative content analysis as described by Mayring [7] could be utilised. The main reasons described as influential for refusal, both from the entire recruitment encounter and the additional nine phone conversations with GPs are presented as "themes" in narrative form (Additional file 2). These data are of qualitative nature; the order of presentation does neither allow any conclusion regarding hierarchy nor frequency. The rational for refusal encompassed structural reasons (e.g. wrong target group or study duration outlasts existence of practice) on the one hand and reasons with regard to contents on the other hand. Besides that, physicians' considered the workload associated with study participation as too much or were afraid of disruption of practice routine. Some doctors criticised that there were no financial rewards offered, others showed a general lack of interest in research.

## Discussion

The recruitment rates obtained were 1.7% and 4.4% during the first and second recruitment phase respectively. When combining the two phases the overall recruitment rate was 2.0%. Asch et al. [8], who conducted a literature review on problems in recruiting community-based physicians, found participation rates ranging from 2.7% to 91%. However, through personal communication with other researchers we came to realise that there is no common agreement as to where the reporting of a recruitment procedure begins. Thus, depending on whether or not researchers consider the initial contacting of physicians as part of the recruitment process, quite different recruitment rates will be obtained. High recruitment rates as described by Asch et al [8] might thus be explained. In the following, our recruitment strategy will be discussed in the light of determining factors that are associated with high participation rates [8].

### Recruitment strategy

Recruitment by sending a letter of introduction has become an integral part of an overall recruitment plan, followed by a phone call, and a face-to-face meeting or phone discussion [5]. However, according to the lessons learnt from Veitch et al. [9] postal recruitment is not efficient whereas phone recruitment is a lengthy, but effective process. Thus, our recruitment procedure differed in that phone calls were the initial contact made to gain permission to send an information fax. Problems encountered with this procedure were that almost all phone calls were taken by physician assistants who decided upon non-participation without consulting the physician. McBride et al. [10] suggested that on-site practice recruitment meetings improve study participation. However, due to financial constraints this could not be realised. The strategy of seeking endorsement from professional societies was unsuccessful. When comparing recruitment rates of GPs with specialists, the latter obtained higher rates showing greater interest in participation. This is in line with Asch et al. [8] who suggest that specialty influences participation rates with specialists showing greater readiness than GPs. Among the various recruitment strategies for GPs the recruiting of the university's cooperation practices was the most successful. It indicates that GP practices affiliated to the university are more open towards research than others. Generally, it is assumed that the utilisation of pre-existing networks is an important asset in enhancing recruitment rates [8].

### Non-physicians recruiting physicians

It became evident very early in the recruitment process that almost all phone calls were going to be answered by physician assistants. Although they are not decision-makers, they are indispensable gatekeepers in facilitating physicians' contact. Traynor et al. [11] remind us that many practice receptionists are expert at deflecting all but essential calls and actually getting through to the doctor becomes a major hurdle. Thus, in many cases the physician assistant decided upon non-participation without consulting the physician. A possible explanation for this might be that the recruiter was a nurse researcher who at this time did not have the title "doctor". Veitch et al. [9] discovered that receptionists are more likely to put a phone call through if the caller's title is "doctor". In addition, profession-political denial may have played a role (theme 7). Some authors report high participation rates if the "physicians recruiting physicians method" is employed [8,12]. In our study, this could have been overcome by engaging a paid physician recruiter who solicits participation from physicians [13]. However, due to financial constraints this was not feasible.

### Study burden and incentives

Equally important for participation is the study burden and time involved. Considering that practices have little time for non-clinical activities, excessive time commitment on the part of the physicians was avoided (theme 4 & 5). Therefore, the interference with physicians' day-to-day clinical work was kept to a minimum, both in terms of administrative work and organisational matters. Other barriers that make physicians refrain from participating include the obtaining of informed consent and intrusion into the patient-physician relationship [5]. Owing to this, physicians only obtained written agreement on forwarding patient details to the coordinator. The informed consent for study participation was then obtained by the research team. As to the intrusion into the patient-physician relationship, there might be a chance that a nurse specialist unintentionally evokes a feeling of interference (theme 5 & 7). Moreover, physicians might fear external assessment [13]. Furthermore, recruiting patients placed an additional burden on physicians' clinical work (theme 4). According to Langley et al. [14] participation requires incentives, but not necessarily of the financial kind. In their study even fairly substantial participant incentives of $250 did not guarantee high participant rates [8]. Nevertheless, Rosemann & Szecsenyi [2] described financial incentives as essential for research participation among German GPs. In our study physician participation was honoured in two ways. First, small gifts were occasionally sent to our practices. Second, we provided a refresher course on leg ulcer treatment for practice staff at the end of the study. This is in line with Hart et al. [15] who successfully recruited physicians by providing CME credit as compensation (theme 6).

### Motivation for physician participation

Levinson et al. [13] suggested that participation may increase if the research topic is relevant to clinical practice. According to Rosemann & Szecsenyi [2] German GPs seem to be interested mainly in research questions that suitably document the high quality of care delivered to patients. Yet treating leg ulcer patients in Germany is financially unattractive and requires an enormous amount of documentation on the part of the physicians. Thus, leg ulceration in general and our research on enhancing patient's self-care in particular might not be considered interesting despite having obtained GP's approval during the modelling phase (theme 8). Anticipating this, the recruiter made an effort to convince physicians of the trial's merits. However, since the majority of phone calls were taken by physician assistants, physicians' potential suggestions for modifications of the study protocol could not be captured. It also emerged that a number of practices already took part in other research studies and thus were reluctant to participate in yet another (theme 4). Salmon et al. [3] provide another explanation for non-participation: GPs show little interest in evidence-based research projects as they consider clinical practice to be an art rather than a science. According to this view evidence-based medicine - as aimed for in our study - seemed to be incompatible with person-centred care. Furthermore, the attractiveness for physicians to participate in our study might rise with an increasing number of leg ulcer patients being treated in the practice. This would explain why participation rates of GPs were lower than those of specialists. Finally, in addition to published German leg ulcer prevalences [16] the average number of leg ulcer patients being treated per practice was requested prior to recruitment from the university's cooperation practices. Yet, when comparing the number of patients enrolled we realised that physicians greatly overestimated the rate. Lovato et al. [5] describe similar findings whereby the retention rate in the pilot study (2%) was double that of the full-scale study (1%).

### Professional rivalry

German GPs are motivated to participate in research which aims at improving the reputation of family medicine [2]. This might be explained by a long-standing conflict between the GPs and specialists in the German health care system [17]. The introduction of community medical nurses - favoured by politicians in Germany - might foster additional professional rivalry between nurses and GPs [18]. Thus GPs might be reluctant to support the nursing profession as mentioned in some phone calls (theme 7). Moreover, our research activities have resulted in little enhancement of professional respectability for GPs, who already have a relatively low status in the German community care sector as compared to specialists [19]. The fact that specialists may not fear nurse-specialist rivalry might explain - among other things - why specialists had a higher participation rate than GPs. Generally, physicians might prefer their own practice assistants to an external nurse specialist. Apart from that, some of the physicians reported negative experiences with wound management nurses in the past. This might be explained by the fact that in the German health care system service companies care for patients in the community with, for example, leg ulceration, incontinence, stoma and other diseases which require consultation-intensive therapeutic appliances. The aim of such services is to enable patients despite their chronic condition to reintegrate in their everyday life and to assume coordination tasks between clinics, doctors and the health insurance fund. Since such service companies are usually self-financed, these nurses were primarily selling wound care products, which led to a bad reputation among physicians (theme 7). Despite great efforts to convince physicians that our study did not focus on the vending of wound dressings some remained reluctant to participate.

### Ownership of the study

Personal interest in the topic, relevance to one's practice, and "buying in" to the study enhances the research experience of the physician and facilitates conduct of study (theme 8) [20]. In order to increase the physicians' acceptability to participate in the presented study a number of measures were taken in the modelling phase. The study protocol was reviewed with a practising GP, discussed in a quality circle of GPs and thus modified. Nevertheless, it seems as if these measures are not sufficient. It might be that the conception of enhancing self-care did not seem of high enough relevance for physicians to "buy into" the idea. Consequently, future research questions need to be generated straight from front-line physician's requirements, seeking to improve their everyday practice.

### Limitations section

The additional phone conversation appears to have been rather limited with regard to the data collection method employed. The analysis of the data obtained during recruitment calls and retrospective calls by means of content analysis was intended, yet the data gained were too scarce for this to be useful. Instead, in-depth interviews with non-participating health professionals and a rigorous method of data analysis would more likely illuminate reasons for recruitment problems and provide valuable lessons for other researchers. However, this might not be feasible in physician's everyday practice due to ongoing high work loads. Financial constraints were another limitation to the study in that comprehensive pilot testing of the recruitment strategy, on-site practice recruitment meetings and the engagement of a paid physician recruiter could not be materialised. Besides that, more effort could have been made prior to study start-up in order to get physicians even more involved in the conception of the study protocol as well as in the evaluation process of the study.

## Conclusion

Interdisciplinary research studies need especially between the medical and nursing professions careful consideration in the development and tailoring of research designs. Time, money, and effort needed during the planning and recruitment phase of a study must not be underestimated to reduce rates of refusal and lack of initial contact. This is an indispensable precondition for attaining recruitment rates sufficient to reach the study projections. Moreover researchers as well as sponsoring bodies should attach greater importance to the execution of a pilot study which is useful for planning the length of the enrolment period, the number of clinical sites, and the financial commitments required. Another important result of this paper is the higher recruitment rate of specialists compared with the rate of GP's in interdisciplinary clinical trials.

## Competing interests

The authors declare that they have no competing interests.

## Authors' contributions

ORH searched the literature for relevant articles on the topic and drafted the whole manuscript. WS and MAR gave advice on the drafted manuscript. All authors critically read and approved the final manuscript.

## Pre-publication history

The pre-publication history for this paper can be accessed here:

http://www.biomedcentral.com/1471-2288/9/61/prepub

## Supplementary Material

Additional file 1 **Results of both recruitment phases specified according to the various recruitment strategies employed**. The table provides an overview of the results of both recruitment phases specified according to the various recruitment strategies employed. Click here for file

Additional file 2 **Thematic grouping of reasons for non-participation of physicians in a clinical trial**. The box provided shows the thematic grouping of reasons for non-participation of physicians in a clinical trial investigating the impact of a nurse specialist on self-care in leg ulcer patients. Click here for file
