# Supplementary material for: Recruitment rates and reasons for community physicians' non-participation in an interdisciplinary intervention study on leg ulceration
Source: BMC Med Res Methodol. 2009 Aug 14;9:61. doi: 10.1186/1471-2288-9-61 (PMC2733138; doi:10.1186/1471-2288-9-61)
Supplement: Additional file 1 — Results of both recruitment phases specified according to the various recruitment strategies employed. The table provides an overview of the results of both recruitment phases specified according to the various recruitment strategies employed. [file 1471-2288-9-61-S1.doc]

**Additional file 1:** Results of both recruitment phases specified according to the various recruitment strategies employed

|  | Cooperation practices of the university  (GP) | Association of CHI Physicians Westphalia–Lippe  (GP) | Association of CHI Physicians North Rhine  (GP) | Yellow Pages  (GP) | Yellow Pages  (dermatologist) | Yellow Pages  (phlebologist) | Directory of professional society of phlebologists | Practice contact lists from nurse specialist or other physicians  (GP) | **Sum** |
| --- | --- | --- | --- | --- | --- | --- | --- | --- | --- |
| **Recruitment phase 1** | | | | | | | | | |
| No. of practices on contact list | 90 | 72 | 52 | 1249 | 363 | n/a[[1]](#footnote-2) | n/a | 49 | 1875 |
| No. of practices contacted | 87 | 62 | 40 | 1010 | 344 | n/a | n/a | 6 | 1549 |
| No. of faxes sent | 51 | 31 | 28 | 439 | 191 | n/a | n/a | 1 | 741 |
| No. of practice characteristics completed | 19 | 8 | 6 | 43 | 29 | n/a | n/a | 0 | 105 |
| No. of participation agreement sent | 11 | 5 | 4 | 12 | 13 | n/a | n/a | 0 | 45 |
| No. of participating practices | 8 | 1 | 2 | 8 | 7 | n/a | n/a | 0 | **26** |
| **Recruitment phase 2** | | | | | | | | | |
| No. of practices on contact list | n/a | n/a | n/a | 223 | 28 | 21 | 20 | 14 | 306 |
| No. of practices contacted | n/a | n/a | n/a | 199 | 27 | 20 | 19 | 8 | 273 |
| No. of faxes sent | n/a | n/a | n/a | 100 | 20 | 16 | 16 | 3 | 155 |
| No. of participation agreement sent | n/a | n/a | n/a | 11 | 5 | 2 | 6 | 0 | 24 |
| No. of participating practices | n/a | n/a | n/a | 4 | 2 | 2 | 4 | 0 | **12** |
| **Recruitment rate**  **according to strategy** | **9.1%** | **1.6%** | **5.0%** | **1.0%** | **2.4%** | **10.0%** | **21.0%** | **0%** |  |

1. n/a = not applicable [↑](#footnote-ref-2)
