# Supplementary material for: Recruitment rates and reasons for community physicians' non-participation in an interdisciplinary intervention study on leg ulceration
Source: BMC Med Res Methodol. 2009 Aug 14;9:61. doi: 10.1186/1471-2288-9-61 (PMC2733138; doi:10.1186/1471-2288-9-61)
Supplement: Additional file 2 — Thematic grouping of reasons for non-participation of physicians in a clinical trial. The box provided shows the thematic grouping of reasons for non-participation of physicians in a clinical trial investigating the impact of a nurse specialist on self-care in leg ulcer patients. [file 1471-2288-9-61-S2.doc]

**Additional file 2:** Thematic grouping of reasons for non-participation of physicians in a clinical trial investigating the impact of a nurse specialist on self-care in leg ulcer patients

| **Wrong target group (1)**   - Physician is mainly psychotherapeutic oriented (=no leg ulcer patients) - Physician is consultant at a hospital (=no office-based physician) - Specialisation in infectious diseases/gynaecology (=no leg ulcer patients) - No leg ulcer patients as regular part of practice - Private practice   **Study duration outlasts existence of practice (2)**   - Liquidation of medical practice - Female doctor is going to take pregnancy leave within study duration - Physician is going to retire within study duration - Physician is going to relocate within study duration - Office remodelling   **Existing routine does not need Tandem practice model (3)**   - Established a system similar to our concept - Physician directly refers leg ulcer patients to medical specialist - Patients are well-cared for already - Cooperation with external wound manager/ diabetologist/ special nurse - Service company takes care of leg ulcer patients   **Team resource exhaustion (4)**   - Limited resources to devote to the project/Insufficient office staff - No time/time pressure/lack of time available to physicians to participate in research/Study is too time-consuming - Participation in another study - Doctor is part of a group practice in which not all practice colleagues were interested in participating   **Office routine (5)**   - Concern about disruption of office routine caused by the study   **Lack of monetary incentives (6)**   - Participation does not generate financial revenue   **Profession-political denial (7)**   - Physicians were dismissive of the nurse specialist as a concept - Nurse specialists were seen as vendors for expensive wound dressings   **Practitioners' research reluctance (8)**   - “Survey fatigue” - Lack of interest to participate in research |
| --- |
